# Treatment patterns, clinical outcomes and health care costs associated with her2-positive breast cancer with central nervous system metastases: a French multicentre observational study

**DOI:** 10.1186/1472-6963-13-456

**Published:** 2013-10-31

**Authors:** Sandrine Baffert, Paul Cottu, Youlia M Kirova, Florence Mercier, Cécile Simondi, Thomas Bachelot, Emilie Le Rhun, Christelle Levy, Maya Gutierrez, Nicolas Madranges, Cristian Moldovan, Bruno Coudert, Dominique Spaëth, Daniel Serin, François-Emery Cotté, Laure Benjamin, Cathie Maillard, Sabine Laulhere-Vigneau, Isabelle Durand-Zaleski

**Affiliations:** 1 Department of Public Health, Health Economics unit, Institut Curie, 26 rue d’Ulm, Paris 75005, France; 2 Department of Medical Oncology, Institut Curie, Paris, France; 3 Department of Radiation Oncology, Institut Curie, Paris, France; 4 Stat Process, Port-Mort, France; 5 Department of Clinical Research, Institut Curie, Paris, France; 6 Department of Medical Oncology, Centre Léon Bérard, Lyon, France; 7 Neurology, Medical Oncology Department, Oscar Lambret Center, Lille, France and Neuro-oncology, University Hospital, Lille, France; 8 Department of Medical Oncology, Centre François Baclesse, Caen, France; 9 Department of Medical Oncology, Institut Curie, Saint-Cloud, France; 10 Department of Medical Oncology, Institut Bergonié, Bordeaux, France; 11 Department of Medical Oncology, Centre Henri Becquerel, Rouen, France; 12 Department of Medical Oncology, Centre JF Leclerc, Dijon, France; 13 Department of Medical Oncology, Centre d’Oncologie de Gentilly, Nancy, France; 14 Department of Medical Oncology, Institut Sainte Catherine, Avignon, France; 15 Department of Pharmaco-Epidemiology and Health Outcomes Research, GlaxoSmithKline, Marly-le-Roi, France; 16 Ceri Medical, Garches, France; 17 Hôpital Henri-Mondor, Créteil, France

**Keywords:** Costs and cost analysis, Cerb-2, HER2-positive, Breast cancer, Brain metastases, Health care costs, Treatment pattern

## Abstract

**Background:**

The population of patients with human epidermal growth factor receptor 2 (HER2)-positive breast cancer (BC) who develop central nervous system (CNS) metastases is growing. Treatment strategies in this population are highly diverse. The objective of the study was to assess health care costs for the management of HER2 positive BC with CNS metastases.

**Methods:**

This multicentre, retrospective, observational study was conducted on HER2-positive BC patients diagnosed with CNS metastases between 2006 and 2008. Data were extracted from patient medical records to estimate health care resource use. A partitioned estimator was used to adjust censoring costs by use of the Kaplan-Meier survival estimate.

**Results:**

218 patients were included and costs were estimated for 200 patients. The median time to detection of CNS metastases was 37.6 months. The first metastatic event involved the CNS in 39 patients, and this was the unique first metastatic site in 31 of these patients. Two years following diagnosis of CNS metastases, 70.3% of patients had died. The mean *per capita* cost of HER2-positive BC with CNS metastases in the first year following diagnosis was €35,735 [95% CI: 31,716-39,898]. The proportion of costs attributed to expensive drugs and those arising from hospitalisation were in the same range.

**Conclusion:**

A range of individualised disease management strategies are used in HER2-positive BC patients with CNS metastases and the treatments used in the first months following diagnosis are expensive. The understanding of cost drivers may help optimise healthcare expenditure and inform the development of appropriate prevention policies.

## Background

In 2011, an estimated 53,000 new cases of breast cancer (BC) were diagnosed and almost 11,500 women died from BC in France [1]. About 5%-15% of new cases of BC are diagnosed at the metastatic stage, when the estimated five-year survival rate is 13% [1]. The apparent incidence of central nervous system (CNS) metastases in BC is increasing [2]. This may be due in part to improved imaging and earlier detection of CNS lesions and in part to the availability of more effective systemic treatments that allow more patients to live long enough to develop CNS metastases [3,4]. In general, survival for BC patients with CNS metastases is poor, with one-year survival of approximately 20% [2]. Compared with patients without CNS metastases, patients with CNS metastases tend to be younger and more likely to have hormone receptor-negative disease and a higher disease burden [5,6].

Over-expression of the gene encoding human epidermal growth factor receptor 2 (HER2) plays an important role in the pathogenesis of certain types of BC [7]. Positive HER2 status is associated with poor prognosis and a higher incidence of CNS metastases in BC. HER2-positive tumours may have a biological predisposition to metastasise within the CNS and about 40% of patients with brain metastases reported in published case series have HER2-positive BC [2,8]. However, despite these initial poor global prognostic features, patients with HER2-positive disease seem to have a better short-term prognosis at the onset of CNS metastases than HER2-negative patients. The development of anti-HER2 targeted therapies such as trastuzumab and lapatinib combined with chemotherapy has significantly improved survival in HER2-positive advanced BC patients [9-12]. Indeed, it has been shown that combination of trastuzumab with standard chemotherapy is associated with significantly improved survival following development of CNS metastases in BC patients compared to patients receiving standard chemotherapy only [5]. This has encouraged systematic screening of HER2 status in patients with BC. In the case series above [2,8], many patients were responding to trastuzumab or had stable systemic disease when they developed brain metastases. However, like many other targeted therapies and cytotoxic chemotherapies, trastuzumab does not easily cross the intact blood–brain barrier [13]. This may lead to sanctuarisation of CNS metastases within the nervous system, allowing them to develop independently of the quality of peripheral disease control.

The management of HER2-positive BC with CNS metastases is based on diversified, non-standardised and increasingly innovative and expensive pharmacological treatments, including targeted therapies, as well as radiotherapy and surgery [8,14]. The costs, risks and benefits of such approaches remain poorly characterised. However, healthcare cost assessment is necessary for such emerging innovative and expensive targeted therapies, particularly in the context of cost-containment initiatives implemented by health authorities in many countries. For example, in France, the recent Program of Social Security funding emphasised the need for input from economic studies to facilitate the decision process relating to reimbursement of health technologies.

Individual current cost data on HER2-positive BC with CNS metastases are limited and published series have mainly reported costs of advanced BC, regardless of their subtype [15-17]. Economic data on healthcare costs can be derived from several sources, including analysis of prescription claims databases, observational studies of patient cohorts and modelling approaches. Each approach has its own advantages and limitations, although cohort studies are thought to provide the most exhaustive information, and represent the only approach that allows costs to be matched to outcomes on an individual patient basis.

The objective of the present study was to describe treatment patterns and health care costs associated with CNS metastases in HER2-positive BC patients over a two-year follow-up period.

## Methods

### Study design

We conducted a multicentre, retrospective, observational study of HER2-positive BC patients diagnosed with CNS metastases. All consecutive patients fulfilling the eligibility criteria were included. Women with HER2-positive BC who had been newly diagnosed with CNS metastases between January 2006 and December 2008 and who were over 18 years of age were eligible. Patients with multiple tumours were excluded. CNS metastases were classified as initial site of relapse or as secondary metastases. Both metastases in the brain parenchyma and leptomeningeal metastases were considered as CNS metastases.

### Study centres

Patients were recruited in ten French cancer centres, selected according to their medical activity (number of patients currently treated) and their compliance with international guidelines for the management of BC and determination of HER2 status [18]. The case-mix of centres was chosen to represent the current standard management of BC in France. In all participating centres, healthcare was funded and reimbursed in the same standardised way though the prospective payment system of the French National Health Service.

### Patient selection

Patients were selected using the disease coding of the ICD 10 (International Classification of Diseases, tenth revision), which is systematically assigned to all patient records in French hospitals. Inpatient stays coded as breast cancer (C50) with brain metastases (C79.3) were selected. Individual data concerning initial diagnosis, distant and CNS relapses, treatments received and examinations performed, clinical outcomes and complications following the CNS event and admissions were collected. Data were collected retrospectively from the hospital database and all information relating to use of healthcare resources was documented for the two years following the date of diagnosis of CNS metastases, or until death if this occurred within two years.

### Clinical outcomes

The clinical outcomes evaluated were overall survival and time to reach Graded Prognostic Assessment (GPA) scores [19-21] of 1–2, 2.5-3 and 3.5-4.

### Identification and allocation of costs

Pricing of health services in France is determined at the national level by a Prospective Payment System. Under this system, the income of each hospital is linked directly to the number and type of patients treated, classified in terms of diagnosis-related groups (DRGs). The classification system used in France was inspired by the US Health Care Financing Group classification, but adapted to the French healthcare system. Assignment of patients to DRGs is based on the primary diagnosis. Data on age, length of stay and mode of discharge (death, transfer) are used to define case severity. The DRG prices (tariffs) for each service are set annually based on average costs and are applicable countrywide. In addition, a restricted list of expensive drugs is reimbursed over and above these standard tariffs.

In the present study, direct medical costs were estimated from the French Health Insurance perspective on the basis of DRG official tariffs and expensive innovative drug tariffs. Costs were estimated from the total mean dose received during each treatment course, the number of courses and the mean price per dose. Costs were expressed in Euros based on 2007 tariffs.

### Statistical analysis

Socio-demographic and clinical variables were compared using the *χ*^2^ test or Fisher’s exact test for categorical variables and *ANOVA* or rank-*ANOVA* for continuous variables. Survival was estimated using the Kaplan-Meier method and the log-rank test. Costs were described in terms of mean values with confidence intervals. The standard deviation of the mean cost was estimated using the Bootstrap resampling method. Since costs per treatment cycle could be limited due to incomplete survival, these costs were censored in order to estimate actual cost expenditure as accurately as possible [22,23]. Costs were adjusted by the Kaplan-Meier estimate for survival to generate a monthly cost, which was used to estimate total costs. All tests were two-sided with a limit of significance of 5%. Data analyses were performed with the SAS software (version 9.2, SAS Institute Inc, USA).

### Ethics

This study was a retrospective observational study that did not modify medical care for people entering the study. For this reason, no written consent form or ethics committee approval was required. Patients were notified by newsletter that the study was being carried out. This study protocol and access to individual patient data were approved by the CCTIRS (*Comité consultatif sur le traitement de l’information en matière de recherche dans le domaine de la santé*, Authorisation N°10-111) and the *Commission Nationale Informatique et Libertés* (CNIL: French Information Technology and Privacy Commission, Authorisation N°2010-060).

## Results

### Patient characteristics

A total of 218 patients were enrolled in the study and direct medical costs were estimated on 200 patients for whom exhaustive economic data and DRGs were available. The clinical characteristics of the 218 patients enrolled are presented in Table 1. Median age was 51 years at the time of initial diagnosis of BC and 54 years at the time of diagnosis of CNS metastases. The median time to first metastatic relapse at any site was 21.9 months (0–214.7). The median interval between BC diagnosis and detection of CNS metastases was 37.6 months (0–286.5). The initial metastatic event involved the CNS in 39 patients (17.4%), this being the unique first metastatic site in 31 patients. Carcinomatous meningitis was present in 28 patients (13%). Prior to diagnosis of CNS metastases, all patients had received trastuzumab-based therapies and 21 had received lapatinib-based therapies.

**Table 1:** Clinical characteristics of the study population (N = 218 patients)

**Initial diagnosis**	
Median age at BC diagnosis (years, range)	51 (24-81)
Invasive ductal carcinoma (patients, %)	202/218 (92.7%)
Median tumour size (mm, range)	25 mm (1-200)*
Presence of lymph node involvement (evaluable tumours, %)	72/134 evaluable tumours (53.7%)
Oestrogen receptor-positive (patients, %)	93/192* (48.4%)
Progesterone receptor-positive (patients, %)	60/164* (36.6%)
**Treatments before CNS metastases**	
Chemotherapy before CNS metastases (patients, %)	210/218 (96.3%)
HER2-targeted therapy before CNS metastases (patients, %)	197/218 (90.4%)
Bevacizumab before CNS metastases (patients, %)	3/218 (1.4%)
Hormone therapy before CNS metastases (patients, %) (ER and PR positive at initial diagnosis N = 110 patients)	82/110 (74.5%)
Local radiation therapy before CNS metastases (patients, %)	159/218 (72.9%)
**Metastatic disease**	
Median time to first metastatic relapse, any site (months, range)	21.9 months (0-214.7)
Median time between initial BC diagnosis and CNS metastasis (months, range)	37.6 months (0-286.5)
Median time to first relapse and CNS metastasis (months, range)	12.9 months (0-283.7)
CNS metastasis at first relapse (patients, %)	39/217 (18%)
Unique first site metastasis	31/217 (14.3%)
Carcinomatous meningitis	28/216 (13%)
CNS metastasis as secondary site (associated with other metastases)	167/218 (76.6%)
Other main metastatic sites at CNS metastasis diagnosis (n = 180)	
Bone	111/178 (62.4%)
Lung/pleura	86/178 (48.3%)
Lymph nodes	101/178 (56.7%)
Liver	34/178 (19.1%)
Median interval between CNS metastasis and other metastases	5.5 months (0.1-29)
**Survival following CNS metastasis**	
Median survival for study population (months, range)	13.1 months (11-15.8)
Median survival for CNS metastasis as first site of relapse	14.2 months (10.6-22.2)
Median survival for secondary CNS metastasis (associated with other metastases)	13 months (10.5-15.8)
Death at 2 years (Kaplan-Meier estimate)	70.27
Median time to GPA threshold 1-2/2.5-3/3.5-4 patients	
Score 1 - 2	10 months
Score 2.5 - 3	12 months
Score 3.5 - 4	24 months

*Data was missing for some patients. BC: breast cancer; CNS: central nervous system; GPA: Graded Prognostic Assessment.

### Clinical outcomes

**Figure 1:**
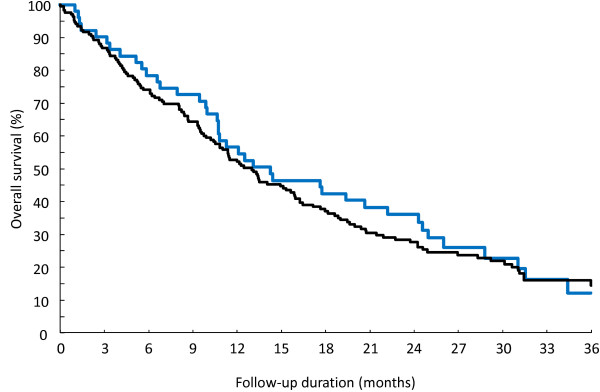
**Overall survival from diagnosis of CNS metastases.** Black: patients with brain metastases as first relapse; blue: patients with secondary metastases.

Median overall survival from diagnosis of CNS metastasis was 13.1 (11–15.8) months (Figure 1). Two years after diagnosis of CNS metastasis, 70.3% of patients had died (Kaplan-Meier estimate). Death was due to progression of CNS metastases (69.7%), distant metastases (12.7%), both (9.3%) or other unspecified cause (8.1%). The median time to reach GPA scores of 1–2, 2.5-3 and 3.5-4 was 10, 12 and 24 months respectively.

### Health-care resources used after diagnosis of CNS metastases

Complete data on resource utilisation were available for 200 patients. Table 2 presents the health-care resources used after diagnosis of CNS metastases. Chemotherapy, including targeted therapy, and radiotherapy were the treatments most frequently used. Whenever significant progression of the disease was observed, a new line (*ie* a new treatment regimen) of chemotherapy was introduced. Up to seven lines of cytotoxic chemotherapy (median: two lines) were given in 185 patients (84.8%) after diagnosis of CNS metastasis. Use of trastuzumab or lapatinib-based therapy decreased from 83.4% for patients on first-line treatment (N = 175) to 48.1% for patients on their fifth line of treatment or more (N = 27). Trastuzumab was most frequently used as a first-line therapy and lapatinib as a second-line therapy. Chemotherapy was mainly delivered during outpatient hospitalisation. Radiotherapy was administered to 200 patients (91.7%), and included whole brain radiotherapy (77.3%), stereotactic radiosurgery (9.6%) or both (6%). Patients received one course of radiotherapy (86.8%), two courses of radiotherapy (12.5%) or a second session of stereotactic radiosurgery (1%). The median number of fractions per patient was ten. Radiation therapy sessions were delivered on an outpatient hospitalisation for 80% (177/229 treatments) and during inpatient admission for 22.7% (52/229) with a median length of stay of thirteen days. Neurosurgery was performed in only 29 patients (13.3%) and the median duration of inpatient admission was seven days. 72.9% (159/218) of patients required an emergency visit at least once during the follow-up period. Patients had 1 to 10 hospital stays either in the index hospital which had diagnosed the CNS metastases or in another establishment. The median length of stay was nine days.

**Table 2:** Health-care resources used after diagnosis of CNS diagnosis

**Chemotherapy including targeted therapy**	**185/218 (84.8%)**			
**Use of trastuzumab- or lapatinib- based therapies**	Trastuzumab	Lapatinib	Both	All
1st line (n = 175)	118 (67.4%)	26 (14.9%)	2 (1.1%)	146 (83.4%)
2nd line (n = 127)	63 (49.6%)	34 (26.8%)	1 (0.8%)	98 (77.2%)
3rd line (n = 81)	39 (48.1%)	15 (18.5%)	2 (2.5%)	56 (69.1%)
4th line (n = 48)	23 (47.9%)	9 (18.8%)	2 (4.2%)	34 (70.9%)
5th line and more (n = 27)	7 (25.9%)	6 (22.2%)	None	13 (48.1%)
**Radiotherapy**	200/218 (91.7%)			
Whole brain radiotherapy only	166/218 (77.3%)			
1 cycle radiotherapy	173/218 (86.8%)			
2 cycles radiotherapy (SRS boost or EBRT)	25/218 (12.5%)			
Second SRS	2/218 (1.0%)			
SRS only	21/218 (9.6%)			
Both (whole-brain radiotherapy and SRS)	13/218 (6.0%)			
Whole-brain radiotherapy 2D	96/193 (49.7%)			
Whole-brain radiotherapy 3D	97/193 (50.3%)			
Median fractions per patient	10 (1–28)			
**Neurosurgery**	29/218 (13.3%)			
**Emergency unit visits**	159/218 (73%)			
Mean length of stay	14.9 days (95% - CI 13.2-16.7)			
Median length of hospital stay (days, range)	9 days (range 1–221)			
Mean number of complications leading to hospitalisation	0.44 (95/218)			

Data are presented as number of patients (%). EBRT: external beam radiotherapy; SRS: stereotactic radiosurgery; 2D: two-dimensional; 3D: three dimensional.

### Health care costs

**Figure 2:**
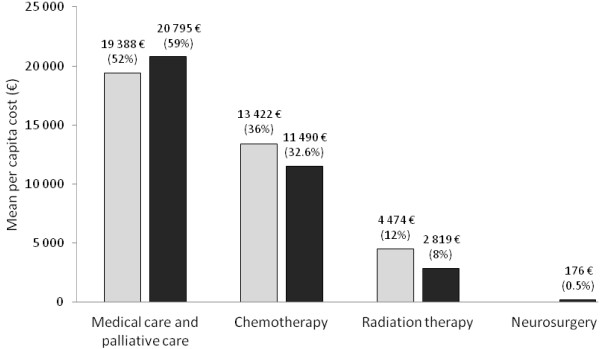
**Distribution of medical costs of patients with CNS metastases over the two year follow-up period.** Open columns: patients with brain metastases as first relapse; filled columns: patients with secondary metastases. Percentages are calculated with respect to total direct medical costs.

Health care costs decreased during the two-year follow-up period, while cost variability increased (Table 3), with the majority of costs concentrated within the first year of management of metastatic disease. The estimated mean treatment cost of HER2-positive BC with CNS metastases was €35,735 per patient [95% CI, 31,716 to 39,898] in the first year following diagnosis of CNS metastasis and €28,939 [95% CI, 22,540 to 35,722] in the second year. Health care costs between patients with CNS metastases as first site of relapse and patients with secondary CNS metastases did not differ significantly at one year after diagnosis of CNS metastasis (€37,284 vs. €35,246, p = 0.73). The distribution of costs by type of resource use is presented in Figure 2 for patients with CNS metastases as first site of relapse and patients with secondary CNS metastases. The proportion of costs attributed to inpatient hospitalisations was similar to those attributed to expensive drugs irrespective of follow-up duration (€18,135 per patient for hospitalisations vs. €17,599 per patient for expensive drugs after one year of follow-up; corresponding to 50.8% and 49.2% of total costs respectively). Medical care and palliative care were the largest cost drivers contributing to 55% of the total inpatient admission cost while chemotherapy and radiation therapy contributed to 35% and 10% of the inpatient admission costs respectively.

**Table 3:** Total mean cost and kaplan-meier estimate per 6-month period (2 years follow-up)

	**First site of relapse**		**Secondary site**	
	**Kaplan-Meier estimate (%)**	**Mean cost (€) [95 CI% (bootstrap)]**	**Kaplan-Meier estimate (%)**	**Mean cost (€) [95 CI% (bootstrap)]**
0-6 months	78.4%	20 227.39 [15 514.81; 25 342.19]	74.0%	19 141.01 [16 885.80; 21 401.47]
7-12 months	56.6%	18 103.14 [12 747.91; 23 712.83]	52.1%	17 150.03 [14 187.45; 20 139.93]
13-18 months	42.4%	19 173.93 [11 648.09; 27 510.13]	37.1%	14 125.19 [10 996.51; 17 530.20]
19-24 months	36.2%	14 376.70 [7 348.29; 22 564.50]	27.7%	14 774.77 [10 117.74; 20 096.10]

## Discussion and conclusions

This retrospective observational study has described treatment patterns, survival and health care resource utilisation for 200 HER2-positive BC patients with CNS metastases treated in ten representative French cancer centres. The clinical characteristics of the observed patients are consistent with those of recently published series on HER2-positive BC with CNS metastases in terms of metastasis characteristics, overall survival from metastatic BC diagnosis, time from metastatic BC diagnosis to development of CNS metastases and treatments after diagnosis of CNS metastases [5,24].

In our study, costs were assessed by site of relapse (CNS metastasis as first relapse or CNS metastasis as secondary site, associated with other metastases). We also considered the line of cytotoxic therapy and the use of targeted therapies specific for HER2. The most frequent treatment approach offered to patients with HER2-positive BC with CNS metastases in this population was chemotherapy, followed by radiotherapy. Chemotherapy was the most important source of cost, comparable to hospitalisation costs. The weight of chemotherapy in expenditure for BC patients underlines the importance of further clinical research to identify the most effective chemotherapeutic treatment regimens for this population of patients. Given the diversity of treatment strategies used in BC with CNS metastases, and our findings showing that chemotherapy is both costly and frequently associated with poor clinical outcome, it seems important to test and validate different treatment algorithms under real-world conditions. This would provide a better estimate of the relative cost-effectiveness of different treatment options for BC with CNS metastases.

In addition, the poor outcomes and high treatment costs observed in these BC patients with CNS metastases suggest a need to diagnose and treat these metastases as early as possible, and before neurological signs appear, in order to optimise the chances of therapeutic success. However, more proactive screening for CNS metastases and earlier treatment may paradoxically generate higher costs of care due to improved life expectancy and longer duration of therapy. Such increased costs would, however, be expected to be partially offset by a better quality of life. Specialist care structures for patients with CNS metastases would also be useful in order to teach patients to recognise and cope with neurological complications with the goal of improving quality of life for patients and their entourage and of limiting emergency department visits and hospitalisations.

We found that the mean cost in the first year following diagnosis was 35,735€ per patient [95% CI: 31,716-39,898]. This estimate appears to be in the same range as those reported in previously published studies, although comparisons should be interpreted with caution due to potential differences in the study population, the range of costs considered and the method of analysis. A small retrospective study in 47 HER2-positive metastatic BC patients in France [25] reported total per-patient direct yearly costs from diagnosis to first metastatic progression of €39,607 for those patients who received trastuzumab. Data from a single-centre, retrospective, observational study of 131 HER2-positive metastatic BC patients receiving trastuzumab in France reported overall annual direct costs per year of €47,832, costs being driven primarily by the costs of trastuzumab (44%) and hospitalisation (41%) [26]. Data from Sweden also indicate that patients with HER2-positive tumours incur higher cost than HER2-negative patients, probably due to the higher cost of targeted therapies, and also to radiotherapy [16].

Further information on the costs of metastatic BC is available from insurance claims databases. A recent French national database analysis estimated the mean annual cost of BC with brain metastases at €29,995 per patient treated with trastuzumab, and assumed to be HER2-positive [27]. In a database analysis from the USA, the direct costs of brain metastases secondary to BC, included medication, outpatient care and inpatient admissions were US$60,045 (€50,898) at six months and US$99,899 (€84,681) at 12 months (2006) [28]. The mean total cost for BC patients with brain metastases was more than twice that of patients without brain metastases at 6 and 12 months.

Although claims database analyses enable broad and exhaustive estimates of cost of illness at a national level to be made relatively rapidly and easily, they are less powerful for comparing sub-groups of populations and for matching economic data to clinical outcomes at the individual patient level. In this respect, observational cohort studies present a number of advantages. Although such studies may be time-consuming and require substantial investment of resources, they provide accurate and detailed information on health care consumption at an individual patient level in both hospital and community settings, and allow the reconstitution of the entire healthcare trajectory of individual patient to be reconstituted. Moreover, cohort analyses provide information on costs in relation to survival and other clinical endpoints. In particular, the influence of potentially different prognosis between sub-groups on health-care consumption can be assessed. Such data are important to collect in order to refine modelling studies of healthcare costs which are critical to inform decision-making with regard to healthcare resource allocation. In France, the recent commitment to including economic assessments into decision-making relating to the drug reimbursement process makes observational cohort studies particularly relevant.

The findings of this study suggest several avenues for future research into the management of BC with CNS metastases in everyday care. These include comparison of costs and outcomes associated with different treatment strategies, which may well not be the same in everyday care than in clinical trials where patients are selected and treatment adherence is optimized. Moreover, the economic and clinical implications of early detection of CNS metastases also need to be evaluated. In addition, it would be of interest to investigate patient-and disease- related determinants of cost and outcome in BC with CNS metastases.

This study has a number of limitations, notably those common to all retrospective observational studies. In particular, the method of selecting patients on the basis of ICD codes may not have allowed exhaustive recruitment due to incomplete or erroneous information in the patient records. We also limited our study to treatments received in the treating centre, which may have led to an underestimation of total costs. In particular, we noticed that surgical interventions were not often performed in the treating centre and are thus not included in the cost estimation. Finally, we used Bang and Tsiatis’ method to handle cost-censored data using the Kaplan-Meier estimator, which may introduce error due to the presence of outliers. This is because the weight attributed to patients incurring very high or low costs will increase over the follow-up period, assuming that they survive.

Our analysis is limited to direct medical costs viewed from the health insurance perspective. Information on non-medical costs such as ambulance transportation and indirect costs such as loss of productivity were not collected since they are not documented in the patient records. Moreover, information on loss of productivity related to sick leave is difficult to collect retrospectively. In many cases, patients with metastatic BC have to stop working altogether. Previous studies have estimated that direct medical costs constitute only about 40% of the total societal cost of these patients [16,17,29].

In conclusion, this study documents the use of a range of individualised disease management strategies in a national cohort of HER2-positive BC patients with CNS metastases. Expensive targeted therapies and cytotoxic agents appear to be the main cost drivers, together with hospitalisation costs. Identifying cost drivers may help optimise healthcare expenditure and inform the development of cost-effective therapeutic strategies.

## Competing interests

Funding for the study was provided by GlaxoSmithKline, purveyors of lapatinib. LB is an employee of GlaxoSmithKline; FEC was an employee of GlaxoSmithKline at the time of the study; SB, PC, YR, TB and IDZ were members of the steering committee of the study. These members, or their institutions, received emoluments from GlaxoSmithKline in recognition of their participation in the study. FM, director of Stat Process, received funding from GlaxoSmithKline for data collection and analysis. CM and SLV are employees of Ceri Medical, who received funding from GlaxoSmithKline for operational management of the study. TB has received consultancy fees from Novartis, Roche and GlaxoSmithKline, speaker’s fees from Novartis and GlaxoSmithKline and research grants from Novartis and Roche. IDZ has received consultancy and speaker fees from Bristol-Myers Squibb. PC has participated in clinical studies for Sanofi, GlaxoSmithKline and Roche and been a speaker from Roche. ELR has received speaker’s fees from GlaxoSmithKline. The other authors have declared having no conflicts of interest.

## Authors’ contributions

SB, PC, YR, TB and IDZ were involved in the steering committee of the study and contributed to preparing the study protocol, statistical interpretation and manuscript writing with the support of F-E.C, LB and Ceri Medical. FM performed all statistical analyses. CS, CM and SLV contributed to study monitoring. ELR, CL, MG, NM, CM, BC, DS and DS contributed to the study as principal investigators of each participating centre. All authors reviewed and approved the manuscript.

## Pre-publication history

The pre-publication history for this paper can be accessed here:

http://www.biomedcentral.com/1472-6963/13/456/prepub
